# A Network for Advancing Prevention and Treatment of Infections Among Immunocompromised Individuals

**DOI:** 10.1001/jamanetworkopen.2025.28383

**Published:** 2025-08-01

**Authors:** Joshua A. Hill, Steven A. Pergam, Natasha B. Halasa, Deepali Kumar, Lindsey R. Baden, Michael J. Boeckh

**Affiliations:** Fred Hutchinson Cancer Center, Seattle, Washington, University of Washington School of Medicine, Seattle; Fred Hutchinson Cancer Center, Seattle, Washington, University of Washington School of Medicine, Seattle; Vanderbilt University Medical Center, Nashville, Tennessee; University Health Network, Toronto, Ontario, Canada; Brigham and Women’s Hospital, Boston, Massachusetts, Harvard University, Cambridge, Massachusetts; Fred Hutchinson Cancer Center, Seattle, Washington, University of Washington School of Medicine, Seattle

## Abstract

**IMPORTANCE:**

Immunocompromised individuals are a large and growing population who are at increased risk for infectious diseases. There has and continues to be a lack of focus on clinical trials to establish the safety and efficacy of therapies for infectious diseases in immunocompromised patients. The establishment of a US-based clinical trial network to improve the study and subsequent implementation of therapies and strategies to treat and prevent infections in immunocompromised individuals would address this gap in research infrastructure and jumpstart public and private investment.

**OBSERVATIONS:**

A national interdisciplinary meeting was convened on September 10, 2024, in Bethesda, Maryland, to discuss the outsized impact of infectious diseases in immunocompromised individuals and to identify the primary gaps and opportunities for clinical trials in this population. Approaches to achieve this goal include obtaining dedicated funding and support through public-private partnerships to establish alignment and feasibility for high-priority areas of research. This article outlines the relevance of this work; ongoing efforts to collaborate with the National Institutes of Health, US Congress, industry, and philanthropy to obtain funding for mutually beneficial outcomes; the network structure; and perspectives from clinicians, regulatory agencies, the pharmaceutical industry, and patients.

**CONCLUSIONS AND RELEVANCE:**

There is a dearth of evidence to support the use of many therapies for infectious diseases in immunocompromised individuals, which has substantial impact at the individual and societal level. A multipronged approach to improve integration of, and funding for, rigorous research in this population into the core priorities of the public and private sectors could address important public health gaps by developing evidence-based guidance to protect a vulnerable community.

## Introduction

### Infectious Diseases Among Immunocompromised Individuals

**Figure 1.**
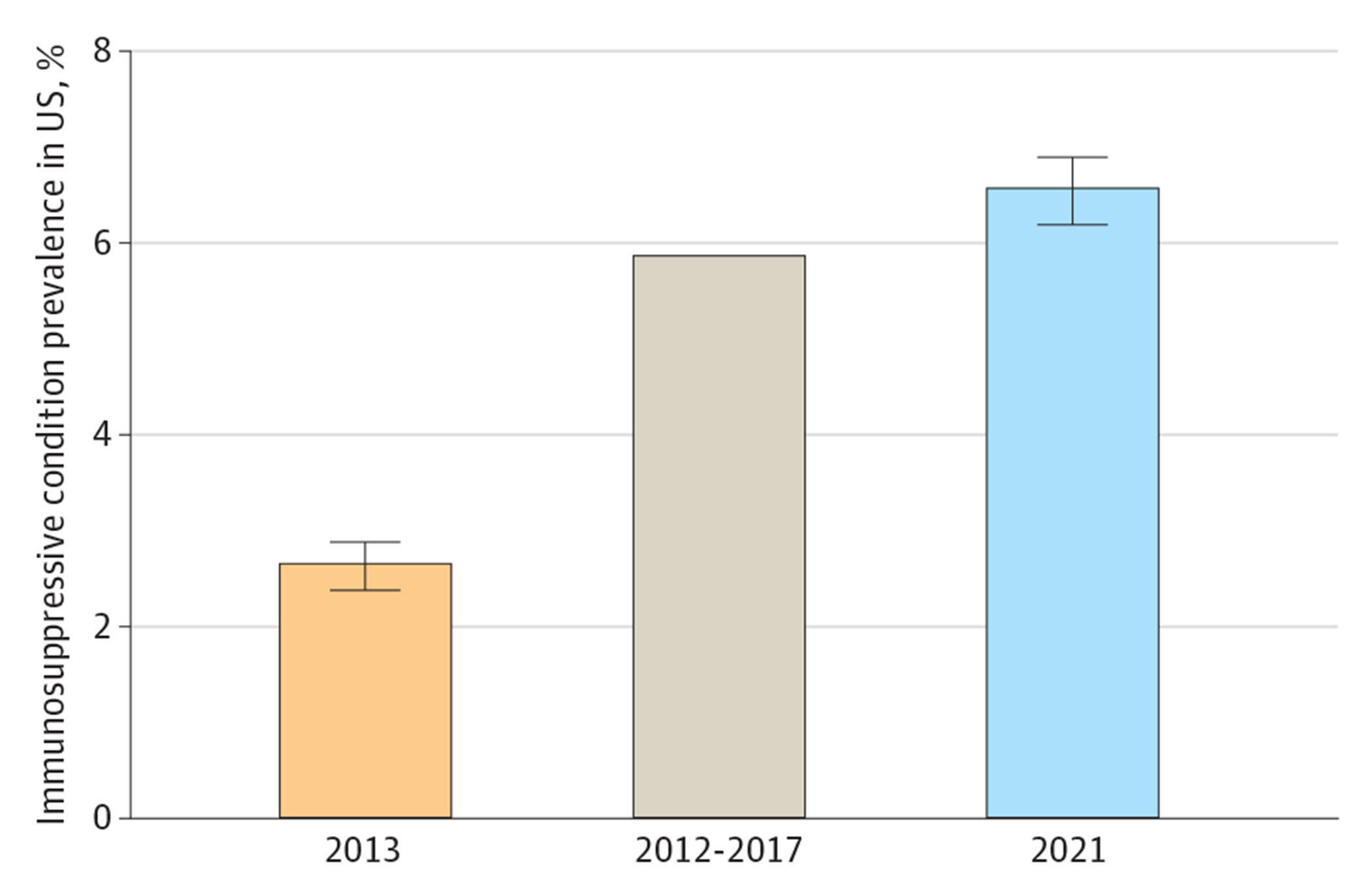
The Immunocompromised Population in the United States Recent estimates indicate that as much as 6.6% of the US population is immunocompromised and has increased over time, representing more than 20 million people in the United States alone. Data for 2013 is from Harpaz et al,^13^ 2016; for 2012 to 2017, Patel et al,^14^ 2020; and for 2021, Martinson and Lapham,^15^ 2024.

Immunocompromised individuals are at high risk for morbidity and mortality due to common and opportunistic infections.^1,2^ The term *immunocompromised* refers to a broad and heterogenous population with varying underlying diseases or treatments as broadly described in Table 1.^3^ In particular, people receiving treatment for cancer, recipients of a hematopoietic cell transplant (HCT) and solid organ transplant (SOT), and people with autoimmune diseases requiring certain types of immunosuppressive therapies are considered to be at moderate to high risk for infections. In studies of long-term survivors after HCT or SOT, patients contracted vaccine-preventable infections at rates up to 30 times higher than the general population.^4-10^ With nearly 12 000 allogeneic HCTs and more than 40 000 SOTs annually in the United States alone,^11,12^ there is a large and growing population at high risk for vaccine-preventable infections. These numbers are substantially higher when extrapolated to the broad scope of increasing medical conditions that require immunosuppressive therapies, which are currently estimated to impact at least 6.6% of the US population and are increasing over time (Figure 1).^13-15^ Importantly, this population has disproportionately higher health care resource utilization.^14^

Immunocompromised individuals also play critical roles in the spread and evolution of certain pathogens due to prolonged shedding and may be a source of viral variants and transmission chains.^16-18^ Prolonged shedding in the presence of drug pressure may also lead to drug resistance.^19^ As an increasingly immunocompromised populace meets a heightened frequency of infectious disease outbreaks and threats on a global scale,^20^ this societal issue is increasingly urgent to address. We are concurrently in a period of rapid advances in immunotherapeutic strategies to enhance immunity, with unprecedented opportunities to prevent and treat infectious diseases.^21-24^ However, existing National Institutes of Health (NIH)–funded networks do not have a scientific focus to address these questions in immunocompromised individuals, and industry-sponsored research in this context is limited. For this reason, substantial uncertainty remains about the optimal timing, dosing, duration, combination, and effectiveness of preventative vaccines, monoclonal antibodies (mAbs), small molecule drugs, and cellular therapies in immunocompromised individuals.^25-31^

### Gaps and Opportunities for Trials Among Immunocompromised Individuals

Despite awareness of this vulnerable population who experience most of the morbidity from infections, particularly due to viral infections, such as influenza and SARS-CoV-2,^2^ there has been a global lack of focus on clinical trials among immunocompromised patients. For instance, their exclusion from primary SARS-CoV-2 vaccine and drug trials led to a lack of evidence-based guidance for these patients and their health care practitioners.^19,32^ A similar gap has emerged for respiratory syncytial virus (RSV) vaccines, for which only limited data are available in immunocompromised patients despite commercial availability of 3 different types of vaccines.^33,34^ The use of many vaccines and mAbs in immunocompromised patients has either not been rigorously studied or evaluated only much later compared with the general population.^30,35-37^ The reasons for this are multifactorial and include challenges in clinical trial enrollment, toxic effects assessment, and drug interactions.^32^ These perceived barriers are addressable issues. For example, developing partnerships with community organizations to engage underrepresented populations and creating patient-centric outreach campaigns can facilitate improved trial enrollment and retention.^32^

Notably, there is an extensive body of work that can be done to address many needs in immunocompromised individuals with vaccines, mAbs, and drugs that are currently available, in development, or in need of development (eFigure in the Supplement), if appropriate infrastructure were in place. However, a fundamental barrier to this work is the fragmentation in funding, in large part due to the heterogeneity in immunocompromised patient populations and the interdisciplinary nature of support for research in this space. Improved integration of and focus on this vulnerable population in the core research priorities of the public and private sector could address important public health gaps, protect a vulnerable community, and jumpstart scientific innovation likely to benefit all people.

## Methods

To engage a diverse group of perspectives in constructive discussion about the importance of and approaches to improved evidence generation for infectious disease management in immunocompromised patients, we convened a multistakeholder meeting in Bethesda, Maryland, on September 10, 2024. A planning committee was formed to collaboratively develop an agenda and potential participants based on geographic representation across the United States, relevant scientific and clinical expertise, and involvement in decisions impacting regulatory and funding decisions for immunocompromised or related populations. The target attendance was approximately 50 to 75 participants to allow for diversity of perspectives while maintaining a constructive working group atmosphere.

## Results

### Developing a Network to Study Treatment and Prevention of Infections Among Immunocompromised Individuals

The workshop was attended by 68 people, including representatives from 17 academic institutions, 7 governmental organizations, 6 professional or patient advocacy organizations, and 7 pharmaceutical companies (eTable 1 in the Supplement). During the meeting, we reviewed the history and development of highly successful networks funded by the NIH, such as the HIV Vaccine Trials Network (HVTN) and the Antibacterial Resistance Leadership Group (ARLG), and public-private partnerships, such as the National Cancer Institute Serological Sciences Network for COVID-19 (SeroNet). There was consensus that none of the existing networks could incorporate the proposed work. We also discussed the formation of the Blood and Marrow Transplant Clinical Trials Network (BMT-CTN), prompted by similar challenges over 2 decades ago in the field of transplantation. After convening a multistakeholder NIH-sponsored meeting like the workshop described here, the NIH released a request for applications (RFA) for a large collaborative award to establish a national network to accelerate research in HCT.^38^ This investment catalyzed many practice-changing studies and created a sustainable and adaptable infrastructure for ongoing work that can rise to meet the occasion when new developments or disruptions (eg, COVID-19) occur.^39,40^

It was clear that fragmentation in funding and prolonged timelines from conception to completion using current grant mechanisms for infectious disease studies in immunocompromised patient populations is severely limiting timely implementation of studies that can have substantial public health benefit. Importantly, it was noted that there is not fragmentation in clinical trial sites or investigators caring for the relevant populations. Thus, there was significant interest across all stakeholders to see public investment to support the genesis of the proposed network, ideally across multiple institutes at the NIH and with opportunities for additional investment from the private sector. Such models have been developed by the BMT-CTN, HVTN, and others, in which substantial public and private support enable sustainability and opportunity to conduct highly efficient and impactful studies.^39,41^ Partnerships with clinical research organizations allow for additional opportunities to engage with industry. To advocate for and obtain the necessary funding, discussions have been initiated and are ongoing with relevant institutes at the NIH, members of the US Congress (for potential authorization and appropriation bills), industry, and fundraising partners. The challenges and potential solutions enabled by a clinical trials network for infectious disease studies in immunocompromised individuals are summarized in Table 2.

### Network Structure

To work toward this vision, 3 initial goals were discussed. The first is to establish a consortium of centers to accelerate implementation of standardized clinical trials designed to assess therapies to prevent and treat infections in patients with moderate-to-high risk (Table 1). The second goal is to establish centralized statistical and data management, laboratory, and operational cores to improve standardization and efficiency of network-sponsored studies. In parallel, a third goal is to develop high-priority clinical trials as detailed in eTable 2 in the Supplement. Fundamentally, this work is aimed to improve therapeutic development for infectious diseases in immunocompromised individuals and generate or provide support for the development of evidence-based guidelines and support tools for patients and health care practitioners. Additionally, it would provide an avenue for novel bench-to-bedside innovations, establish a platform for future efforts to scale to broader groups of immunocompromised patients, and be in place for rapid deployment when the next epidemic arrives.

**Figure 2.**
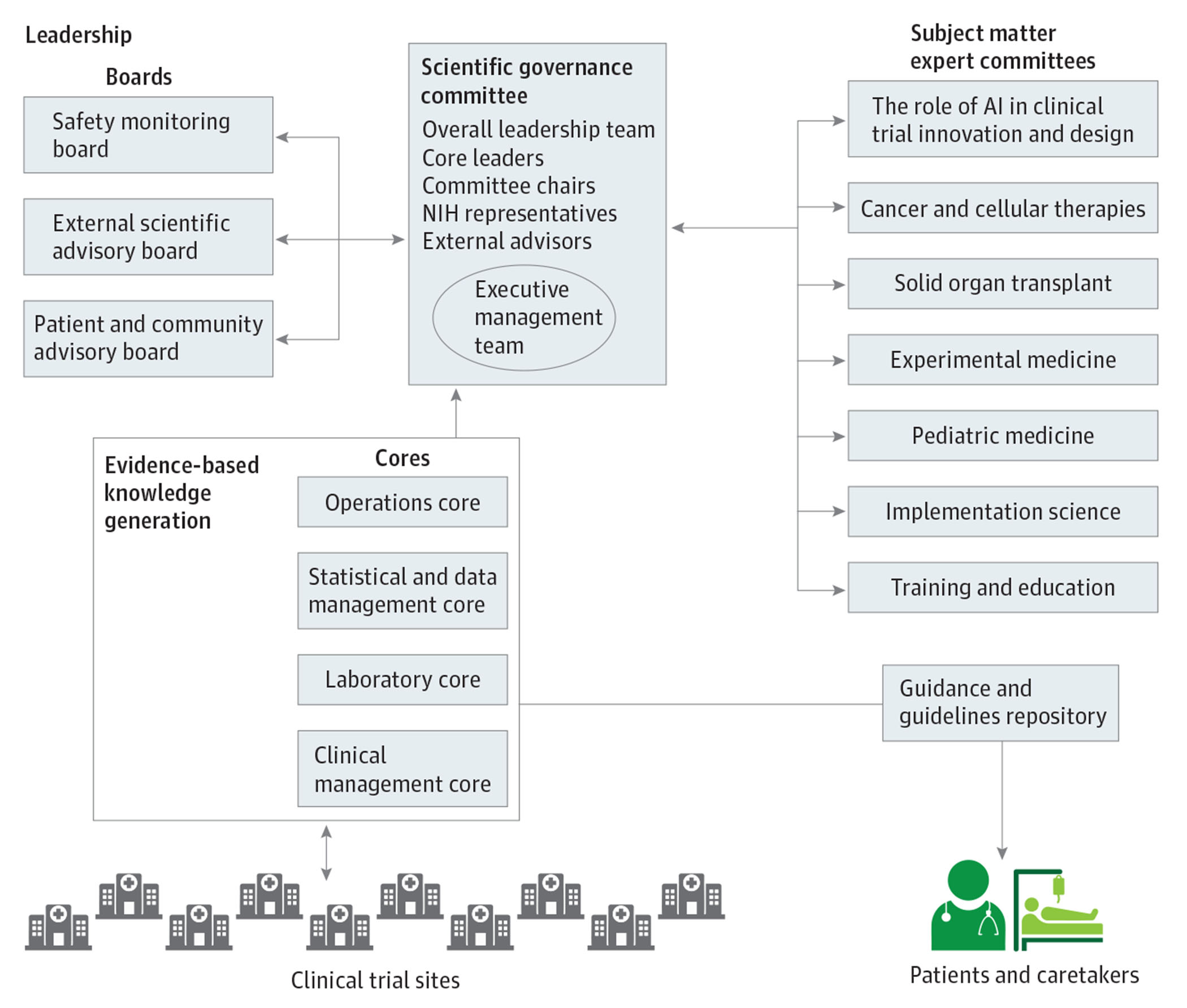
Proposed Structure and Output of a Dedicated Network Focusing on Clinical Trials of Therapeutics to Prevent and Treat Infectious Diseases Among Immunocompromised Patients With High Risk AI indicates artificial intelligence; NIH, National Institutes of Health.

The network structure proposed below is designed to encourage participatory science, integration of diverse ideas into the network’s scientific agenda, and innovative approaches (eg, artificial intelligence [AI]) to improve clinical trial conduct (Figure 2). In this section, we outline the vision for a fully developed network, recognizing that a phased approach will be necessary.

#### Scientific Governance Committee

The central hub of the network will be composed of the Scientific Governance Committee (SGC), which will provide scientific, administrative, fiscal, and programmatic leadership for the network. The SGC will consist of the overall leadership team, the leaders of each core, committee chairs, NIH representatives, and external advisors. Diverse representation will be emphasized. The Executive Management Team will be a group of individuals within the SGC to provide day-to-day leadership and decision-making. A conflict of interest policy will be in place to ensure that the development and implementation of the research agenda is free from bias.

#### Statistical and Data Management and Operations Cores

The Statistical and Data Management Core (SDMC) will support protocol development, statistical analysis plans, data management, the external safety monitoring board (SMB), and publications. The Operations Core will be responsible for project management and administration, including study development, conduct, and monitoring; financial management; regulatory support; and administrative support. There will be a distinct focus on leveraging and developing tools utilizing AI to improve efficiencies and modernize clinical trial conduct where applicable.

#### Laboratory Core

The Laboratory Core will provide relevant expertise, leadership, guidance, and support for laboratory aspects of the network’s studies. Based on the potential breadth of studies and pathogens that may be targeted, this will require collaborations with public and private partners for state-of-the-art laboratory testing support. Responsibilities will include standardization of sample collection, study-specific assays (when relevant), processing, and maintenance of a biorepository.

#### Subject Matter Expert Committees

Multiple committees will serve as forums for ideas and expertise that inform the network’s scientific focus. There will be2 committees that focus on the distinct populations representing the initial focus of the network, including the Cancer and Cellular Therapies Steering Committee and the SOT Committees. An Experimental Medicine Committee will support development of innovative therapeutic strategies with early-phase clinical trials. Additional cross-cutting committees will inform key considerations applicable to all studies, including pediatrics and implementation science, to improve the availability and adoption of clinical advances to affected individuals. A committee focused on AI in clinical trial innovation and design will be tasked with optimizing trial design and conduct using strategies developed by the JAMA Summit series,^42^ for example. This work would include strategies for rapid protocol development, implementation, and iterative assessment to tailor best approaches for larger trials^43^ in addition to leveraging AI throughout the process of designing and running clinical trials.^44^ Finally, a Training and Education Committee will play a critical role in mentorship and training physicians and researchers focused on immunocompromised patients. Integrating learning, exposure to clinical trials, and connection to thought leaders can provide a sustainable means for perpetuating applicable research and the network.^45^

#### Advisory Boards

The external Scientific Advisory Board will be responsible for providing feedback and guidance on scientific and operational issues as well as network performance. This group will be composed of members from diverse backgrounds, including academic institutions, the private sector, the NIH, the US Centers for Disease Control and Prevention, the US Food and Drug Administration (FDA), and patient advocates. The SMB will consist of a panel of academic investigators and nonscientific community reviewers to provide independent evaluation of the safety of interventional trials. A separate Patient and Community Engagement Advisory Board will bring the patient voice into all aspects of network priorities and facilitate conveying information back to affected communities.

#### Participating Sites

To expediently conduct clinical trials, we envision initial partnerships with 20 academic institutions with high patient volumes in the targeted populations at moderate-to-high infection risk (patients with cancer, cellular therapy recipients, and SOT recipients) (Table 1). Geographically distributed sites across North America will be competitively selected based on experience, track record, infrastructure, and evidence of dedicated engagement in relevant areas of research by the institution and associated investigative teams. Master agreements will be established with each participating site, and as new protocols are launched, riders will be executed on existing agreements to save time and expense.

### Stakeholder Discussions

#### Patients

During the meeting, there was robust discussion about the patient perspective for the proposed work from patient representatives and organizations. Beyond the direct impact of a new medical diagnosis on an individual’s life, learning about being immunocompromised due to the disease, treatment for the disease, or both results in additional stress. Being immunocompromised can have large impacts on one’s daily life, such as having to avoid social situations, travel, or hobbies to limit risk for certain infections. Furthermore, the challenges of such identity shifts can be exacerbated by the uncertainty around efficacy and availability of prevention and treatment options for both common and emerging infections. Patients and other attendees noted the lack of focus on immunosuppressed patient populations in clinical trials and emphasized the need for public-private partnerships to improve patient engagement in designing future studies. There was also discussion of the critical role of patient and community advisors in helping with advocacy and recruitment.

#### Regulatory Agencies

There are broad ranging implications of the lack of safety and efficacy data for vaccines and other therapies in special populations with unique risks, especially regarding access. After approval is granted by the FDA, insurers use recommendations from the national Advisory Committee on Immunization Practices to determine which vaccines they will cover. Approvals and guidance specific to special populations, such as the immunocompromised, are infrequent due to limited data, resulting in challenges for access and utilization. Off-label administration of products (eg, RSV vaccines in high-risk patients <18 years old) may require significant out-of-pocket expense, if accessible at all, adding to financial toxicity and inequities in health care already faced by these populations.^46,47^

To stimulate investment in evidence generation in this special population by the private sector, we reviewed aspects of the regulatory process for therapeutic approvals at the FDA as it pertains to immunocompromised individuals. There is currently no statutory requirement to generate vaccine safety and effectiveness data in this patient population, although the European Medicines Agency recently published guidelines to encourage sponsors to conduct clinical trials of new vaccines in immunocompromised individuals, either before or after initial authorization.^48^ Additionally, no specific regulatory incentive programs exist to accelerate research in this area. As a result, evidence generation in immunocompromised patients is infrequently pursued. To address these gaps, we discussed opportunities to leverage existing FDA expedited programs, such as Fast Track and Priority Review designations, that could be applied to immunocompromised populations and stimulate private sector interest. A new policy, such as the Best Pharmaceuticals for Children Act that was enacted to incentivize the study and labeling of medications for pediatric use, should be explored for immunocompromised groups. There was clear consensus in a shared public health goal of safe and effective vaccines and other therapies across stakeholders. To ensure uptake of new therapies and engender the trust of health care practitioners and patients alike, it is critical that data are generated to accurately inform prescribers and patients of the benefits and risks associated with existing and new treatments.

#### Industry

From the industry perspective, market forces sideline immunocompromised patients absent a commitment to this population and adoption of a patient-centric approach. The group identified a need for evolution in access pathways through improved flexibility or incentives in regulatory approval processes, particularly for rapidly changing and emerging pathogens. Additionally, slow accrual of immunocompromised patients in trials is a major impediment to studying this population. Finally, there was discussion of the importance of de-risking investments by industry through public-private partnerships.

#### Health Care Practitioners

Considering the challenges discussed in evidence generation for treatment and prevention of infectious diseases in immunocompromised individuals, where investment by industry at an early stage in therapeutic development is relatively limited, a proposal for the establishment of a national clinical trial network was discussed as a foundational step forward. There was collective agreement that a dedicated network of clinical sites with high volumes of relevant patients, centralized expertise, and existing infrastructure to rapidly conduct trials in immunocompromised patients could foster prealignment with industry partners on timelines and expectations to de-risk financial investments.

## Conclusions

There are substantial gaps in the established knowledge of how to best deploy existing therapies, and in the infrastructure for testing available and novel therapies, to prevent and treat infections in the steadily growing population of immunocompromised individuals. A coordinated national network focused on this task will facilitate the health care community’s ability to meet this unique moment in medicine by applying rapid developments of new interventions to some of the most vulnerable patients in our society. The envisioned network will establish a critical bulwark against the perfect storm that will yet again emerge with the convergence of a rapidly increasing sector of the population that is immunocompromised alongside mounting global infectious disease threats. Such an infrastructure could provide unique efficiencies and opportunities to conduct practice-changing studies on an accelerated timeline, in addition to an advocacy platform for issues pertaining to regulatory and policy considerations. This community of investigators will enable invaluable opportunities for training the next generation of biomedical scientists and health care professionals with expertise in clinical trial conduct in immunocompromised patients. Realizing this vision of tackling our present-day challenges through a conceptual framework of “roots, not parachutes”^49^ will require dedicated public and private support for a lasting impact.

## Supplementary Material

Supplement **eTable 1.** Institutions and Organizations Represented by Attendees at the September 10, 2024,Workshop
**eTable 2.** Initial Studies That Could Be Considered for Implementation Through the Envisioned Network
**eFigure.** Status of Availability and Evidence for Small Molecule Drugs, Vaccines, Monoclonal Antibodies (mAbs), and Cellular Therapies for Pathogens in Immunocompromised (IC) vs Not IC Individuals

## Figures and Tables

**Table 1.** Proposed Framework for Consideration of Infectious Disease Risk Level Categorization Among Immunocompromised Patient Populations Based on Disease or Treatment^a^

Risk category	Example health condition	Example therapeutics
Higher risk	Hematopoietic cell transplant within 1 y or requiring treatment for graft-vs-host disease Hematologic malignant neoplasm requiring treatment Lung transplant B-cell aplasia based on an absolute CD19^+^ B cell count <20 cells/mm^3^ within 6 mo	B-cell depleting drugs (eg, rituximab) CAR-T cell therapy T-cell depleting therapy in the prior 6 mo (eg, anti-thymocyte globulin)
Moderate risk	Solid organ transplant other than lung Solid tumor on treatment HIV infection with CD4 <200 cells/mm^3^ Primary immunodeficiency resulting in low immunoglobulins or B/T cell defects Autoimmune disease requiring high dose corticosteroids or >1 immunosuppressive drug for disease control	Anthracycline derivatives High-dose corticosteroids defined as >20 mg prednisone or equivalent for >4 wk
Lower risk	HIV infection with CD4 >200 cells/mm^3^ Cirrhosis End stage kidney disease Autoimmune disease not requiring high dose corticosteroids or >1 immunosuppressive drug for disease control	Anti-TNF Corticosteroids <20 mg prednisone or equivalent for <4 wk

Abbreviations: CAR, chimeric antigen receptor; TNF, tumor necrosis factor.

a Categorization may also depend on the pathogen (eg, kidney transplant recipients could be a high-priority population for vaccines and therapies targeting BK virus). Adapted from Bhimraj et al,^3^ 2024.

**Table 2.** Challenges in Evidence Generation for the Prevention and Treatment of Infectious Diseases in Immunocompromised Individuals and Potential Solutions Enabled by a Clinical Trial Network

Challenges and evidence gaps	Potential solutions enabled by a network
There is a growing proportion of society with immunocompromising conditions, resulting in a higher number of individuals at increased risk for infectious disease complications and attendant health care resource utilization Reduced immune responses to routine vaccines underscores the importance of novel approaches to vaccination and alternative treatments (eg, monoclonal antibodies) Immunocompromised individuals are frequently excluded from clinical trials Drug adverse effect profiles may be different in immunocompromised individuals, requiring independent assessment of safety of new therapeutics FDA approvals and expert guidance specific to immunocompromised populations are limited due to lack of evidence-based data Limited regulatory requirements and incentives to catalyze industry investment in therapeutic development for infectious diseases in immunocompromised individuals Funding for studies in immunocompromised individuals is fragmented due to heterogeneity of diseases and treatments	Centralized scientific leadership and expertise to guide and implement high priority clinical trials Established network of participating sites with relevant expertise and patient populations Centralized operations to improve standardization and efficiency in clinical trial conduct Partnerships with public and private organizations for collaboration, funding, policy, and advocacy Established infrastructure for responding to changing patient populations (eg, new type of immunosuppressive treatment), novel or emergent pathogens, and new therapeutic modalities Coordinated communication and dissemination of evidence to improve patient care

Abbreviation: FDA, US Food and Drug Administration.

## References

[1] Roberts MB, Fishman JA. Immunosuppressive agents and infectious risk in transplantation: managing the “net state of immunosuppression”. Clin Infect Dis. 2021;73(7):e1302–e1317. doi:10.1093/cid/ciaa1189 32803228 PMC8561260

[2] Leston M, Elson W, Ordóñez-Mena JM, Disparities in COVID-19 mortality amongst the immunosuppressed: a systematic review and meta-analysis for enhanced disease surveillance. J Infect. 2024;88(3):106110. doi:10.1016/j.jinf.2024.01.009 38302061 PMC10943183

[3] Bhimraj A, Falck-Ytter Y, Kim AY, 2024 Clinical practice guideline update by the Infectious Diseases Society of America on the management of COVID-19: anti-SARS-CoV-2 neutralizing antibody pemivibart for pre-exposure prophylaxis. Clin Infect Dis. Published online October 29, 2024. doi:10.1093/cid/ciae435 39471458

[4] Foord AM, Cushing-Haugen KL, Boeckh MJ, Late infectious complications in hematopoietic cell transplantation survivors: a population-based study. Blood Adv. 2020;4(7):1232–1241. doi:10.1182/bloodadvances.2020001470 32227211 PMC7160274

[5] Fishman JA. Infection in organ transplantation. Am J Transplant. 2017;17(4):856–879. doi:10.1111/ajt.14208 28117944

[6] Lee DH, Zuckerman RA; AST Infectious Diseases Community of Practice. Herpes simplex virus infections in solid organ transplantation: guidelines from the American Society of Transplantation Infectious Diseases Community of Practice. Clin Transplant. 2019;33(9):e13526. doi:10.1111/ctr.13526 30859647

[7] Pergam SA, Limaye AP; AST Infectious Diseases Community of Practice. Varicella zoster virus in solid organ transplantation: guidelines from the American Society of Transplantation Infectious Diseases Community of Practice. Clin Transplant. 2019;33(9):e13622. doi:10.1111/ctr.13622 31162727

[8] Razonable RR, Humar A. Cytomegalovirus in solid organ transplant recipients: guidelines of the American Society of Transplantation Infectious Diseases Community of Practice. Clin Transplant. 2019;33(9):e13512. doi:10.1111/ctr.13512 30817026

[9] Manuel O, Estabrook M; American Society of Transplantation Infectious Diseases Community of Practice. RNA respiratory viral infections in solid organ transplant recipients: guidelines from the American Society of Transplantation Infectious Diseases Community of Practice. Clin Transplant. 2019;33(9):e13511. doi:10.1111/ctr.13511 30817023 PMC7162209

[10] So M, Walti L. Challenges of antimicrobial resistance and stewardship in solid organ transplant patients. Curr Infect Dis Rep. 2022;24(5):63–75. doi:10.1007/s11908-022-00778-1 35535263 PMC9055217

[11] Bolon YT, Atshan R, Allbee-Johnson M, Estrada-Merly N, Lee SJ. 2022 US summary slides (PowerPoint). Center for International Blood & Marrow Transplant Research. Accessed July 22, 2025. https://cibmtr.org/CIBMTR/Resources/Summary-Slides-Reports

[12] Schladt DP, Israni AK. OPTN/SRTR 2021 annual data report: introduction. Am J Transplant. 2023;23(2)(suppl 1):S12–S20. doi:10.1016/j.ajt.2023.02.003 37132351

[13] Harpaz R, Dahl RM, Dooling KL. Prevalence of Immunosuppression Among US Adults, 2013. JAMA. 2016;316(23):2547–2548. doi:10.1001/jama.2016.16477 27792809

[14] Patel M, Chen J, Kim S, Analysis of MarketScan data for immunosuppressive conditions and hospitalizations for acute respiratory illness, United States. Emerg Infect Dis. 2020;26(8):1720–1730. doi:10.3201/eid2608.191493 32348234 PMC7392442

[15] Martinson ML, Lapham J. Prevalence of immunosuppression among US adults. JAMA. 2024;331(10):880–882. doi:10.1001/jama.2023.28019 38358771 PMC10870224

[16] Corey L, Beyrer C, Cohen MS, Michael NL, Bedford T, Rolland M. SARS-CoV-2 variants in patients with immunosuppression. N Engl J Med. 2021;385(6):562–566. doi:10.1056/NEJMsb2104756 34347959 PMC8494465

[17] Casadevall A, Focosi D. SARS-CoV-2 variants resistant to monoclonal antibodies in immunocompromised patients constitute a public health concern. J Clin Invest. 2023;133(6):e168603. doi:10.1172/JCI168603 36919696 PMC10014096

[18] Hurt AC, Chotpitayasunondh T, Cox NJ, ; WHO Consultation on Pandemic Influenza A (H1N1) 2009 Virus Resistance to Antivirals. Antiviral resistance during the 2009 influenza A H1N1 pandemic: public health, laboratory, and clinical perspectives. Lancet Infect Dis. 2012;12(3):240–248. doi:10.1016/S1473-3099(11)70318-8 22186145

[19] Renaud C, Boudreault AA, Kuypers J, H275Y mutant pandemic (H1N1) 2009 virus in immunocompromised patients. Emerg Infect Dis. 2011;17(4):653–660. doi:10.3201/eid1704.101429 21470455 PMC3290123

[20] Baker RE, Mahmud AS, Miller IF, Infectious disease in an era of global change. Nat Rev Microbiol. 2022;20(4):193–205. doi:10.1038/s41579-021-00639-z 34646006 PMC8513385

[21] Adigweme I, Yisa M, Ooko M, A measles and rubella vaccine microneedle patch in the Gambia: a phase 1/2, double-blind, double-dummy, randomised, active-controlled, age de-escalation trial. Lancet. 2024;403(10439):1879–1892. doi:10.1016/S0140-6736(24)00532-4 38697170 PMC11099471

[22] Aliahmad P, Miyake-Stoner SJ, Geall AJ, Wang NS. Next generation self-replicating RNA vectors for vaccines and immunotherapies. Cancer Gene Ther. 2023;30(6):785–793. doi:10.1038/s41417-022-00435-8 35194198 PMC8861484

[23] Guo S, Feng J, Li Z, Improved cancer immunotherapy strategies by nanomedicine. Wiley Interdiscip Rev Nanomed Nanobiotechnol. 2023;15(3):e1873. doi:10.1002/wnan.1873 36576112

[24] Lee JH, Sutton HJ, Cottrell CA, Long-primed germinal centres with enduring affinity maturation and clonal migration. Nature. 2022;609(7929):998–1004. doi:10.1038/s41586-022-05216-9 36131022 PMC9491273

[25] Bavaro DF, Diella L, Belati A, Efficacy of remdesivir and neutralizing monoclonal antibodies in monotherapy or combination therapy in reducing the risk of disease progression in elderly or immunocompromised hosts hospitalized for COVID-19: a single center retrospective study. Viruses. 2023;15 (5):1199. doi:10.3390/v15051199 37243285 PMC10222024

[26] Boonyaratanakornkit J, Boeckh M, Waghmare A. Monoclonal antibodies for prophylaxis and treatment of respiratory viral infections. Curr Opin Infect Dis. 2022;35(4):280–287. doi:10.1097/QCO.0000000000000846 35849517

[27] Cabán M, Rodarte JV, Bibby M, Cross-protective antibodies against common endemic respiratory viruses. Nat Commun. 2023;14(1):798. doi:10.1038/s41467-023-36459-3 36781872 PMC9923667

[28] Conway SR, Keller MD, Bollard CM. Cellular therapies for the treatment and prevention of SARS-CoV-2 infection. Blood. 2022;140(3):208–221. doi:10.1182/blood.2021012249 35240679 PMC8896869

[29] Keller MD, Bollard CM. Virus-specific T-cell therapies for patients with primary immune deficiency. Blood. 2020;135(9):620–628. doi:10.1182/blood.2019000924 31942610 PMC7046606

[30] Schaenman J, Avery R. Cowpox to COVID: history of vaccination in the immunocompromised host. Transpl Infect Dis. 2023;25(3):e14051. doi:10.1111/tid.14051 37029494

[31] Singh S, Kumar NK, Dwiwedi P, Monoclonal antibodies: a review. Curr Clin Pharmacol. 2018;13(2):85–99. doi:10.2174/1574884712666170809124728 28799485

[32] Boeckh M, Pergam SA, Limaye AP, Englund J, Corey L, Hill JA. How immunocompromised hosts were left behind in the quest to control the COVID-19 pandemic. Clin Infect Dis. 2024;79(4):1018–1023. doi:10.1093/cid/ciae308 38825885 PMC11478583

[33] Karaba AH, Hage C, Sengsouk I, Antibody response to respiratory syncytial virus vaccination in immunocompromised persons. JAMA. 2025;333(5):429–432. doi:10.1001/jama.2024.25395 39786402 PMC11795327

[34] Bajema KL, Yan L, Li Y, Respiratory syncytial virus vaccine effectiveness among US veterans, September, 2023 to March, 2024: a target trial emulation study. Lancet Infect Dis. 2025;25(6):625–633. doi:10.1016/S1473-3099(24)00796-5 39848264

[35] Andrei G, Snoeck R. Advances and perspectives in the management of Varicella zoster virus infections. Molecules. 2021;26(4):1132. doi:10.3390/molecules26041132 33672709 PMC7924330

[36] Trøseid M, Hentzien M, Ader F, ; EU RESPONSE; COMBINE. Immunocompromised patients have been neglected in COVID-19 trials: a call for action. Clin Microbiol Infect. 2022;28(9):1182–1183. doi:10.1016/j.cmi.2022.05.005 35623577 PMC9130310

[37] Cowan J, Amson A, Christofides A, Chagla Z. Monoclonal antibodies as COVID-19 prophylaxis therapy in immunocompromised patient populations. Int J Infect Dis. 2023;134:228–238. doi:10.1016/j.ijid.2023.06.021 37400053

[38] O’Reilly RJ. Clinical trials of hematopoietic cell transplantations: current needs and future strategies. Biol Blood Marrow Transplant. 2000;6(2):79–89. doi:10.1016/S1083-8791(00)70070-X 10741616

[39] Devine SM, Horowitz MM. Building a fit for purpose clinical trials infrastructure to accelerate the assessment of novel hematopoietic cell transplantation strategies and cellular immunotherapies. J Clin Oncol. 2021;39(5): 534–544. doi:10.1200/JCO.20.01623 33434065 PMC8443822

[40] Steering Committee Of The Blood And Marrow Transplant Clinical Trials Network. The Blood and Marrow Transplant Clinical Trials Network: an effective infrastructure for addressing important issues in hematopoietic cell transplantation. Biol Blood Marrow Transplant. 2016;22(10):1747–1757. doi:10.1016/j.bbmt.2016.07.003 27418009 PMC5027144

[41] Kublin JG, Morgan CA, Day TA, HIV Vaccine Trials Network: activities and achievements of the first decade and beyond. Clin Investig (Lond). 2012;2(3):245–254. doi:10.4155/cli.12.8 PMC3521567 23243491

[42] Bibbins-Domingo K, Angus DC, Park H, Introducing the JAMA Summit. JAMA. 2024;331(17):1451. doi:10.1001/jama.2024.5570

[43] Angus DC, Huang AJ, Lewis RJ, ; JAMA Summit on Clinical Trials Participants. The integration of clinical trials with the practice of medicine: repairing a house divided. JAMA. 2024;332(2):153–162. doi:10.1001/jama.2024.4088 38829654 PMC12045079

[44] Hutson M. How AI is being used to accelerate clinical trials. Nature. 2024;627(8003):S2–S5. doi:10.1038/d41586-024-00753-x 38480968

[45] Phadke VK, Nematollahi S, Steinbrink JM, Defining the landscape of educational experiences in transplant infectious diseases: a national survey of infectious diseases fellows in the United States. Open Forum Infect Dis. 2024;11(9):ofae473. doi:10.1093/ofid/ofae473 39263215 PMC11389608

[46] Yabroff KR, Dowling EC, Guy GP Jr, Financial hardship associated with cancer in the United States: findings from a population-based sample of adult cancer survivors. J Clin Oncol. 2016;34(3):259–267. doi:10.1200/JCO.2015.62.0468 26644532 PMC4872019

[47] Bhatia S, Dai C, Hageman L, Financial burden in blood or marrow transplantation survivors during the COVID-19 pandemic: a BMTSS report. J Clin Oncol. 2023;41(5):1011–1022. doi:10.1200/JCO.22.00461 36455192 PMC9928670

[48] European Medicines Agency. Clinical evaluation of new vaccines—scientific guideline. January 8, 2023. Accessed July 23, 2025. https://www.ema.europa.eu/en/clinical-evaluation-new-vaccines-scientific-guideline

[49] Yozwiak NL, Happi CT, Grant DS, Roots, not parachutes: research collaborations combat outbreaks. Cell. 2016;166(1):5–8. doi:10.1016/j.cell.2016.06.029 27368093 PMC5210219

