# Supplementary material for: A Network for Advancing Prevention and Treatment of Infections Among Immunocompromised Individuals
Source: JAMA Netw Open. Author manuscript; Available in PMC 2026 Aug 10. (PMC13456121; doi:10.1001/jamanetworkopen.2025.28383)
Supplement: Supplementary file 1 [file NIHMS2195931-supplement-Supplement.pdf]

Supplemental Online Content

Hill JA, Pergam SA, Halasa NB, Kumar D, Baden LR, Boeckh MJ. A network for advancing prevention and treatment of infections among immunocompromised individuals. *JAMA Netw Open.* 2025;8(8):e2528383. doi:10.1001/jamanetworkopen.2025.28383

eTable 1. Institutions and Organizations Represented by Attendees at the September 10, 2024, Workshop

eTable 2. Initial Studies That Could Be Considered for Implementation Through the Envisioned Network

eFigure. Status of Availability and Evidence for Small Molecule Drugs, Vaccines, Monoclonal Antibodies (mAbs), and Cellular Therapies for Pathogens in Immunocompromised (IC) vs Not IC Individuals

This supplemental material has been provided by the authors to give readers additional information about their work.

© 2025 Hill JA et al. *JAMA Network Open*.

eTable 1. Institutions and Organizations Represented by Attendees at the September 10, 2024, Workshop

**Government**
Frederick National Laboratory for Cancer Research
National Institutes of Health
U.S. Centers for Disease Control and Prevention
U.S. Food & Drug Administration
Walter Reed Army Institute of Research
**Foundations and Patient Advocates**
Chronic Lymphocytic Leukemia (CLL) Society
Fred Hutchinson Cancer Center Patient and Family Engagement Program
Infectious Diseases Society of America
American Society of Transplantation
American Society for Transplantation and Cellular Therapy
National Marrow Donor Program
**Industry**
AstraZeneca
CSL Seqirus
GSK
Leidos
Moderna
Pfizer
Sanofi
**Academia**
Ann & Robert H. Lurie Children's Hospital of Chicago (Chicago, IL)
Brigham and Women's Hospital (Boston, MA)
Cincinnati Children's (Cincinnati, OH)
City of Hope (Duarte, CA)
Dana-Farber Cancer Institute (Boston, MA)
Duke University (Durham, NC)
Emory University (Atlanta, GA)
Fred Hutchinson Cancer Center (Seattle, WA)
Johns Hopkins University (Baltimore, MD)
Massachusetts General Hospital (Boston, MA)
Memorial Sloan Kettering Cancer Center (New York City, NY)
Seattle Children's Hospital (Seattle, WA)
University of Pittsburgh (Pittsburgh, PA)
The University of Texas MD Anderson Cancer Center (Houston, TX)
University of Toronto (Toronto, Canada)
University of Washington (Seattle, WA)
Vanderbilt University Medical Center (Nashville, TN)

© 2025 Hill JA et al. *JAMA Network Open*.

eTable 2. Initial Studies That Could Be Considered for Implementation Through the Envisioned Network

**Clinical Trials and Observational Studies**			
**Product**	**Pathogen target examples**	**Stage**	**Design considerations**
Vaccines	RSV[a], H5N1[a] and other avian influenza viruses, SARS-CoV-2	Phase I – IV	• Interventional trials with investigational and commercially approved products with consideration for changes in dose or frequency<br>• Focus on safety and maximizing immunogenicity<br>• Observational studies of commercially administered products<br>• Conceptual study designs of alternative vaccine administration strategies to increase immunogenicity (e.g., fractionated dosing, microneedle patch delivery)<br>• Master platform type protocols and adaptive designs
Monoclonal antibodies	Respiratory viruses, dsDNA viruses	Phase I – III	• Prophylaxis and treatment designs
Small molecule drugs	Herpesviruses, respiratory viruses, BK virus[a], adenovirus	Phase II – IV	• Preemptive or prophylactic treatment trials in high-risk patients beyond labeled indications and with investigational products<br>• Combination therapies[a]
Cellular therapies	dsDNA viruses, invasive molds	Phase I – III	• Preemptive or prophylactic treatment trials
Diagnostics	Variable	N/A	• Establishing performance characteristics of novel diagnostic assays

**Other Deliverables**
**Description**
• Define standardized adverse event assessment grading, reporting, and stopping criteria for interventional trials in specific immunocompromised patient populations (e.g., HCT versus SOT)

© 2025 Hill JA et al. *JAMA Network Open*.

- Develop standardized clinical trial protocol templates and workflows
- Establish a registry for rare and emerging infections or infections in specific existing or new patient populations (e.g., treated with new chemo-immunotherapy) to support priority trials and trial designs
- Establish a specimen biorepository

dsDNA virus indicates double-stranded DNA viruses such as the herpesviruses, BK virus, and adenovirus.
[a]Indicates pathogens or concepts that are considered higher priority.

© 2025 Hill JA et al. *JAMA Network Open*.

eFigure. Status of Availability and Evidence for Small Molecule Drugs, Vaccines, Monoclonal Antibodies (mAbs), and Cellular Therapies for Pathogens in Immunocompromised (IC) vs Not IC Individuals

Categories	Pathogen(s)	Drugs		Vaccines		mAbs		Cellular Therapies	
		IC	Not IC	IC	Not IC	IC	Not IC	IC	Not IC
Herpesviruses	Herpes simplex (HSV)								
	Varicella zoster (VZV)								
	Epstein-Barr (EBV)					#	#	^	
	Cytomegalovirus (CMV)							#	
	Roseola (HHV-6)	#	#					#	
	Kaposi sarcoma-associated herpesvirus (KSHV; HHV-8)								
Respiratory Viruses	Influenza								
	SARS-CoV-2								
	Respiratory syncytial (RSV)	#	#						
	Common cold coronaviruses (ccCoV)								
	Adenovirus (ADV)							#	
	Parainfluenza (PIV1-4)								
	Human metapneumovirus (HMPV)								
	Rhinovirus (RhV)								
	Enteroviruses								
Hepatic Viruses	Hepatitis A virus (HAV)								
	Hepatitis B virus (HBV)								
	Hepatitis C virus (HCV)								
Gastrointestinal Viruses	Rotavirus (HRV)								
	Norovirus (HNoV)								
Other Viruses	JC virus							#	
	BK virus							#	
	Human papillomavirus (HPV)								
	Parvovirus B19								
Fungal Pathogens	Yeast								
	Mold							#	
	Pneumocystis jirovecii (PJP)								
Bacterial Pathogens	Gram-positive bacteria			*#					
	Gram-negative bacteria								
	C. difficile								

Data and therapy available

Data for immunocompromised individuals unavailable, therapy available

Therapy unavailable

[#]Therapy exists but not approved for this indication

*Data for immunocompromised individuals only available for *S. pneumoniae*

^ Only availble in the European Union

IC - immunocompromised individuals

This figure was generated based on select pathogens (or pathogen groups) of interest in immunocompromised individuals. Annotation and categorization were based on targeted literature review.

IC indicates immunocompromised.

Hyperimmune globulin is available for post-exposure prophylaxis and treatment for select pathogens but is not depicted here. For EBV, available mAbs target CD20 on B cells (i.e., rituximab) and are not pathogen specific.

© 2025 Hill JA et al. *JAMA Network Open*.
